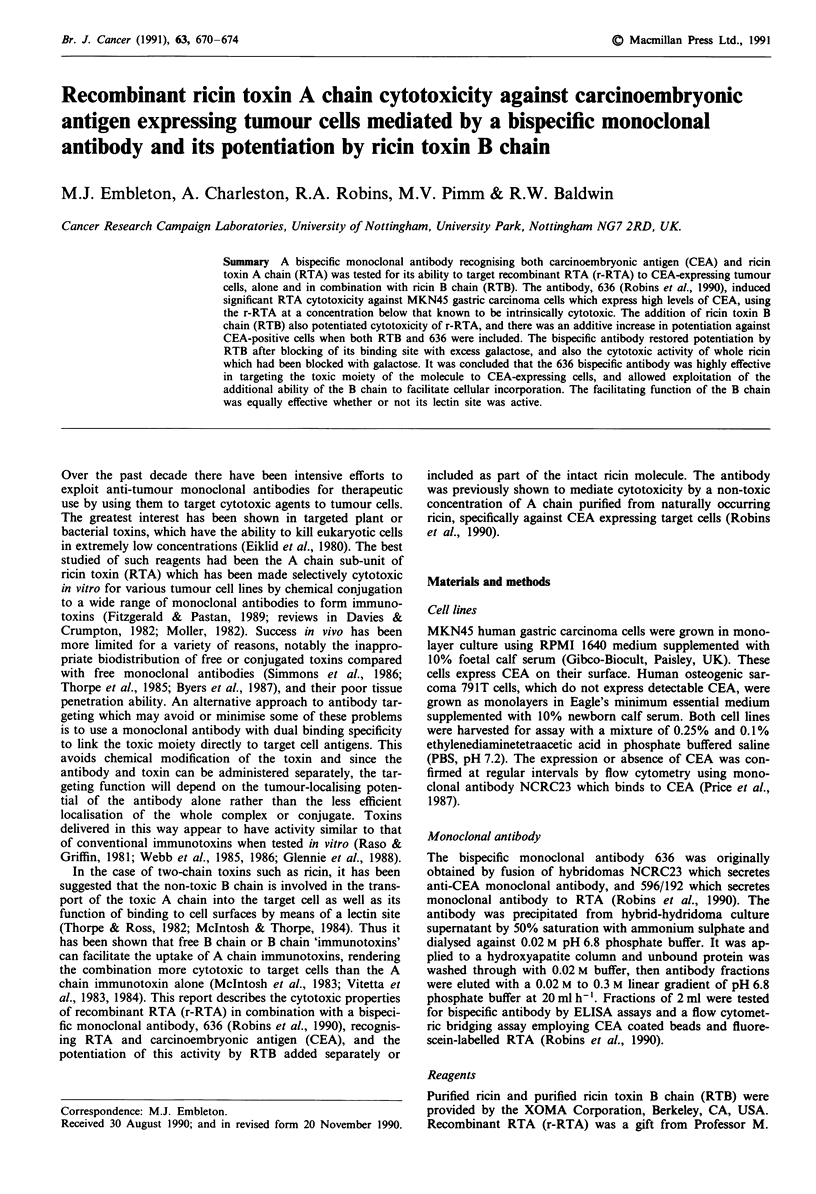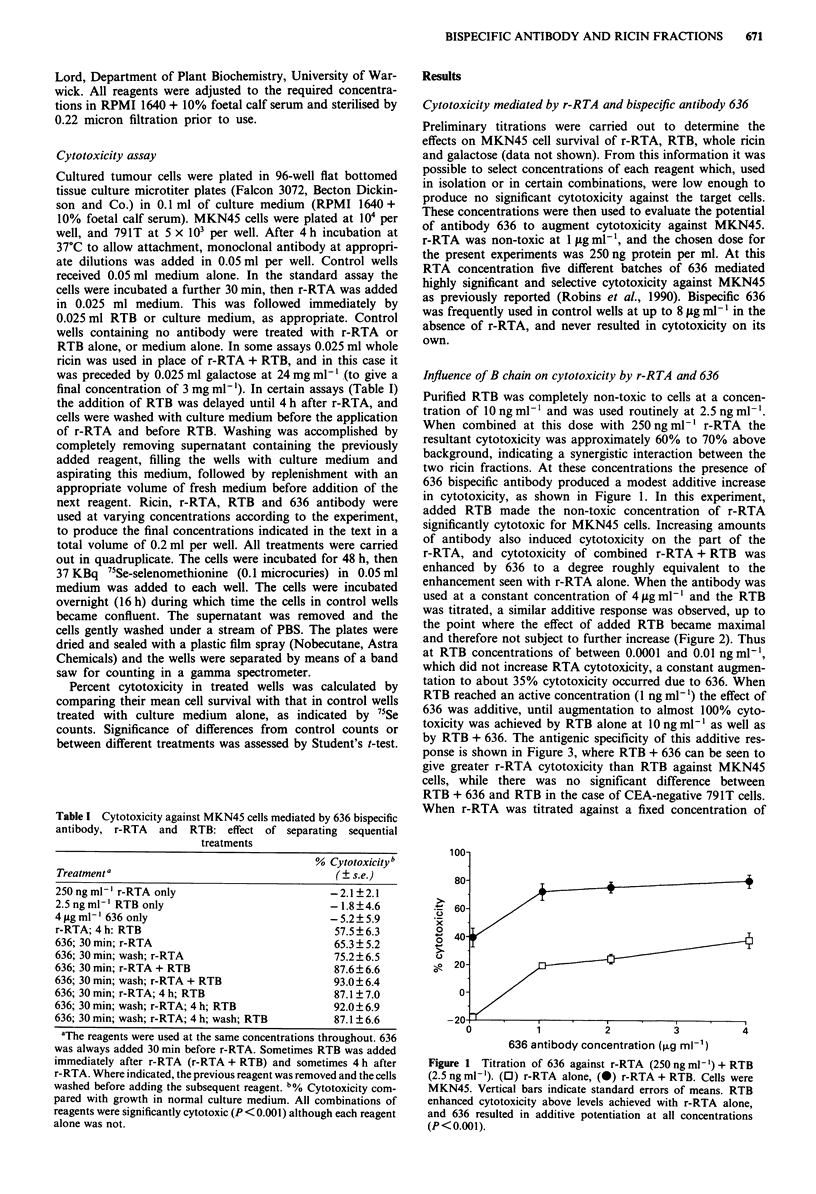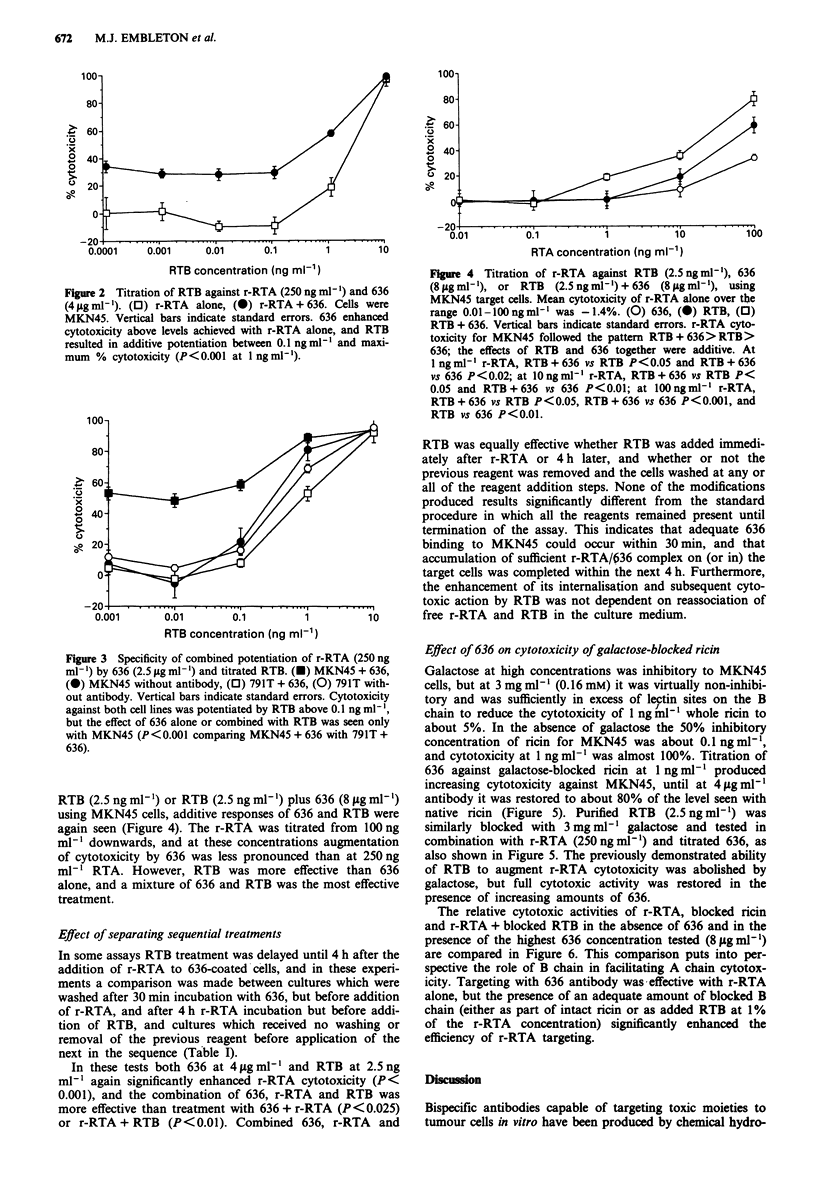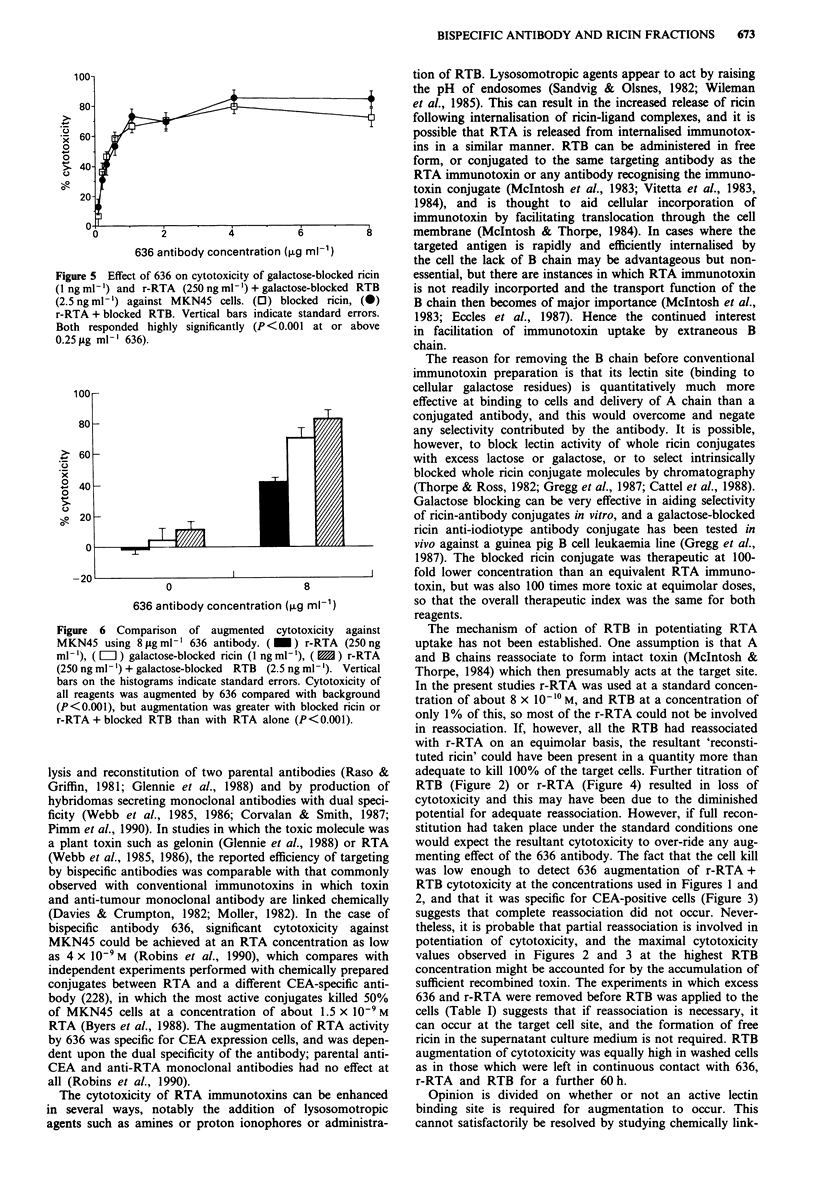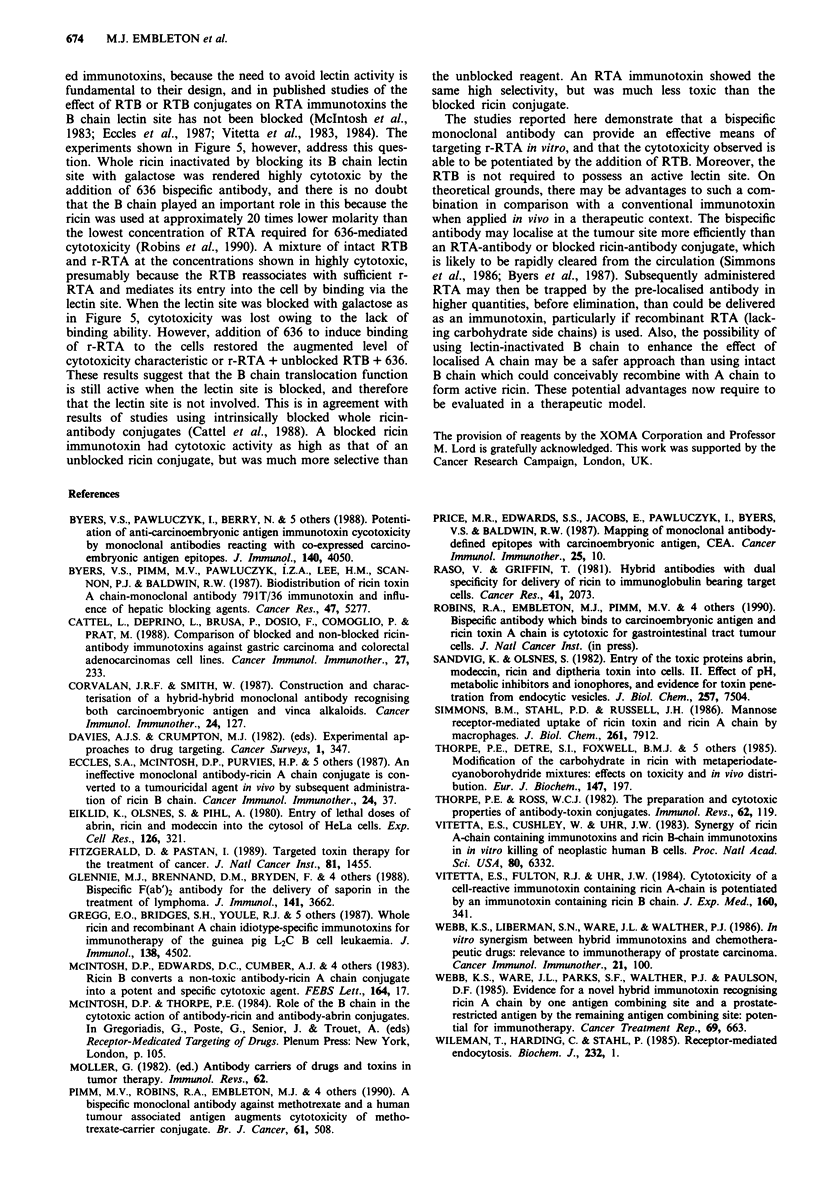# Recombinant ricin toxin A chain cytotoxicity against carcinoembryonic antigen expressing tumour cells mediated by a bispecific monoclonal antibody and its potentiation by ricin toxin B chain.

**DOI:** 10.1038/bjc.1991.153

**Published:** 1991-05

**Authors:** M. J. Embleton, A. Charleston, R. A. Robins, M. V. Pimm, R. W. Baldwin

**Affiliations:** Cancer Research Campaign Laboratories, University of Nottingham, University Park, UK.

## Abstract

A bispecific monoclonal antibody recognising both carcinoembryonic antigen (CEA) and ricin toxin A chain (RTA) was tested for its ability to target recombinant RTA (r-RTA) to CEA-expressing tumour cells, alone and in combination with ricin B chain (RTB). The antibody, 636 (Robins et al., 1990), induced significant RTA cytotoxicity against MKN45 gastric carcinoma cells which express high levels of CEA, using the r-RTA at a concentration below that known to be intrinsically cytotoxic. The addition of ricin toxin B chain (RTB) also potentiated cytotoxicity of r-RTA, and there was an additive increase in potentiation against CEA-positive cells when both RTB and 636 were included. The bispecific antibody restored potentiation by RTB after blocking of its binding site with excess galactose, and also the cytotoxic activity of whole ricin which had been blocked with galactose. It was concluded that the 636 bispecific antibody was highly effective in targeting the toxic moiety of the molecule to CEA-expressing cells, and allowed exploitation of the additional ability of the B chain to facilitate cellular incorporation. The facilitating function of the B chain was equally effective whether or not its lectin site was active.


Br. J. Cancer (1991), 63, 670-674 © Macmillan Press Ltd., 1991

### Recombinant ricin toxin A chain cytotoxicity against carcinoembryonic antigen expressing tumour cells mediated by a bispecific monoclonal antibody and its potentiation by ricin toxin B chain

M.J. Embleton, A. Charleston, R.A. Robins, M.V. Pimm & R.W. Baldwin

*Cancer Research Campaign Laboratories, University of Nottingham, University Park, Nottingham NG7 2RD, UK.*

**Summary** A bispecific monoclonal antibody recognising both carcinoembryonic antigen (CEA) and ricin toxin A chain (RTA) was tested for its ability to target recombinant RTA (r-RTA) to CEA-expressing tumour cells, alone and in combination with ricin B chain (RTB). The antibody, 636 (Robins *et al.*, 1990), induced significant RTA cytotoxicity against MKN45 gastric carcinoma cells which express high levels of CEA, using the r-RTA at a concentration below that known to be intrinsically cytotoxic. The addition of ricin toxin B chain (RTB) also potentiated cytotoxicity of r-RTA, and there was an additive increase in potentiation against CEA-positive cells when both RTB and 636 were included. The bispecific antibody restored potentiation by RTB after blocking of its binding site with excess galactose, and also the cytotoxic activity of whole ricin which had been blocked with galactose. It was concluded that the 636 bispecific antibody was highly effective in targeting the toxic moiety of the molecule to CEA-expressing cells, and allowed exploitation of the additional ability of the B chain to facilitate cellular incorporation. The facilitating function of the B chain was equally effective whether or not its lectin site was active.

Over the past decade there have been intensive efforts to exploit anti-tumour monoclonal antibodies for therapeutic use by using them to target cytotoxic agents to tumour cells. The greatest interest has been shown in targeted plant or bacterial toxins, which have the ability to kill eukaryotic cells in extremely low concentrations (Eiklid *et al.*, 1980). The best studied of such reagents had been the A chain sub-unit of ricin toxin (RTA) which has been made selectively cytotoxic *in vitro* for various tumour cell lines by chemical conjugation to a wide range of monoclonal antibodies to form immunotoxins (Fitzgerald & Pastan, 1989; reviews in Davies & Crumpton, 1982; Moller, 1982). Success *in vivo* has been more limited for a variety of reasons, notably the inappropriate biodistribution of free or conjugated toxins compared with free monoclonal antibodies (Simmons *et al.*, 1986; Thorpe *et al.*, 1985; Byers *et al.*, 1987), and their poor tissue penetration ability. An alternative approach to antibody targeting which may avoid or minimise some of these problems is to use a monoclonal antibody with dual binding specificity to link the toxic moiety directly to target cell antigens. This avoids chemical modification of the toxin and since the antibody and toxin can be administered separately, the targeting function will depend on the tumour-localising potential of the antibody alone rather than the less efficient localisation of the whole complex or conjugate. Toxins delivered in this way appear to have activity similar to that of conventional immunotoxins when tested *in vitro* (Raso & Griffin, 1981; Webb *et al.*, 1985, 1986; Glennie *et al.*, 1988).

In the case of two-chain toxins such as ricin, it has been suggested that the non-toxic B chain is involved in the transport of the toxic A chain into the target cell as well as its function of binding to cell surfaces by means of a lectin site (Thorpe & Ross, 1982; McIntosh & Thorpe, 1984). Thus it has been shown that free B chain or B chain 'immunotoxins' can facilitate the uptake of A chain immunotoxins, rendering the combination more cytotoxic to target cells than the A chain immunotoxin alone (McIntosh *et al.*, 1983; Vitetta *et al.*, 1983, 1984). This report describes the cytotoxic properties of recombinant RTA (r-RTA) in combination with a bispecific monoclonal antibody, 636 (Robins *et al.*, 1990), recognising RTA and carcinoembryonic antigen (CEA), and the potentiation of this activity by RTB added separately or included as part of the intact ricin molecule. The antibody was previously shown to mediate cytotoxicity by a non-toxic concentration of A chain purified from naturally occurring ricin, specifically against CEA expressing target cells (Robins *et al.*, 1990).

### Materials and methods

### *Cell lines*

MKN45 human gastric carcinoma cells were grown in monolayer culture using RPMI 1640 medium supplemented with 10% foetal calf serum (Gibco-Biocult, Paisley, UK). These cells express CEA on their surface. Human osteogenic sarcoma 791T cells, which do not express detectable CEA, were grown as monolayers in Eagle's minimum essential medium supplemented with 10% newborn calf serum. Both cell lines were harvested for assay with a mixture of 0.25% and 0.1% ethylenediaminetetraacetic acid in phosphate buffered saline (PBS, pH 7.2). The expression or absence of CEA was confirmed at regular intervals by flow cytometry using monoclonal antibody NCRC23 which binds to CEA (Price *et al.*, 1987).

### *Monoclonal antibody*

The bispecific monoclonal antibody 636 was originally obtained by fusion of hybridomas NCRC23 which secretes anti-CEA monoclonal antibody, and 596/192 which secretes monoclonal antibody to RTA (Robins *et al.*, 1990). The antibody was precipitated from hybrid-hydridoma culture supernatant by 50% saturation with ammonium sulphate and dialysed against 0.02 M pH 6.8 phosphate buffer. It was applied to a hydroxyapatite column and unbound protein was washed through with 0.02 M buffer, then antibody fractions were eluted with a 0.02 M to 0.3 M linear gradient of pH 6.8 phosphate buffer at 20 ml $h^{-1}$. Fractions of 2 ml were tested for bispecific antibody by ELISA assays and a flow cytometric bridging assay employing CEA coated beads and fluorescein-labelled RTA (Robins *et al.*, 1990).

### *Reagents*

Purified ricin and purified ricin toxin B chain (RTB) were provided by the XOMA Corporation, Berkeley, CA, USA. Recombinant RTA (r-RTA) was a gift from Professor M.

Correspondence: M.J. Embleton.
Received 30 August 1990; and in revised form 20 November 1990.

Lord, Department of Plant Biochemistry, University of Warwick. All reagents were adjusted to the required concentrations in RPMI 1640 + 10% foetal calf serum and sterilised by 0.22 micron filtration prior to use.

*Cytotoxicity assay*

Cultured tumour cells were plated in 96-well flat bottomed tissue culture microtiter plates (Falcon 3072, Becton Dickinson and Co.) in 0.1 ml of culture medium (RPMI 1640 + 10% foetal calf serum). MKN45 cells were plated at $10^4$ per well, and 791T at $5 \times 10^3$ per well. After 4 h incubation at 37°C to allow attachment, monoclonal antibody at appropriate dilutions was added in 0.05 ml per well. Control wells received 0.05 ml medium alone. In the standard assay the cells were incubated a further 30 min, then r-RTA was added in 0.025 ml medium. This was followed immediately by 0.025 ml RTB or culture medium, as appropriate. Control wells containing no antibody were treated with r-RTA or RTB alone, or medium alone. In some assays 0.025 ml whole ricin was used in place of r-RTA + RTB, and in this case it was preceded by 0.025 ml galactose at 24 mg ml$^{-1}$ (to give a final concentration of 3 mg ml$^{-1}$). In certain assays (Table I) the addition of RTB was delayed until 4 h after r-RTA, and cells were washed with culture medium before the application of r-RTA and before RTB. Washing was accomplished by completely removing supernatant containing the previously added reagent, filling the wells with culture medium and aspirating this medium, followed by replenishment with an appropriate volume of fresh medium before addition of the next reagent. Ricin, r-RTA, RTB and 636 antibody were used at varying concentrations according to the experiment, to produce the final concentrations indicated in the text in a total volume of 0.2 ml per well. All treatments were carried out in quadruplicate. The cells were incubated for 48 h, then 37 KBq $^{75}$Se-selenomethionine (0.1 microcuries) in 0.05 ml medium was added to each well. The cells were incubated overnight (16 h) during which time the cells in control wells became confluent. The supernatant was removed and the cells gently washed under a stream of PBS. The plates were dried and sealed with a plastic film spray (Nobecutane, Astra Chemicals) and the wells were separated by means of a band saw for counting in a gamma spectrometer.

Percent cytotoxicity in treated wells was calculated by comparing their mean cell survival with that in control wells treated with culture medium alone, as indicated by $^{75}$Se counts. Significance of differences from control counts or between different treatments was assessed by Student's *t*-test.

**Table I** Cytotoxicity against MKN45 cells mediated by 636 bispecific antibody, r-RTA and RTB: effect of separating sequential treatments

| *Treatment*[a] | *% Cytotoxicity*[b] (± *s.e.*) |
|---|---|
| 250 ng ml$^{-1}$ r-RTA only | −2.1 ± 2.1 |
| 2.5 ng ml$^{-1}$ RTB only | −1.8 ± 4.6 |
| 4 μg ml$^{-1}$ 636 only | −5.2 ± 5.9 |
| r-RTA; 4 h: RTB | 57.5 ± 6.3 |
| 636; 30 min; r-RTA | 65.3 ± 5.2 |
| 636; 30 min; wash; r-RTA | 75.2 ± 6.5 |
| 636; 30 min; r-RTA + RTB | 87.6 ± 6.6 |
| 636; 30 min; wash; r-RTA + RTB | 93.0 ± 6.4 |
| 636; 30 min; r-RTA; 4 h; RTB | 87.1 ± 7.0 |
| 636; 30 min; wash; r-RTA; 4 h; RTB | 92.0 ± 6.9 |
| 636; 30 min; wash; r-RTA; 4 h; wash; RTB | 87.1 ± 6.6 |

[a]The reagents were used at the same concentrations throughout. 636 was always added 30 min before r-RTA. Sometimes RTB was added immediately after r-RTA (r-RTA + RTB) and sometimes 4 h after r-RTA. Where indicated, the previous reagent was removed and the cells washed before adding the subsequent reagent. [b]% Cytotoxicity compared with growth in normal culture medium. All combinations of reagents were significantly cytotoxic ($P < 0.001$) although each reagent alone was not.

### Results

*Cytotoxicity mediated by r-RTA and bispecific antibody 636*

Preliminary titrations were carried out to determine the effects on MKN45 cell survival of r-RTA, RTB, whole ricin and galactose (data not shown). From this information it was possible to select concentrations of each reagent which, used in isolation or in certain combinations, were low enough to produce no significant cytotoxicity against the target cells. These concentrations were then used to evaluate the potential of antibody 636 to augment cytotoxicity against MKN45. r-RTA was non-toxic at 1 μg ml$^{-1}$, and the chosen dose for the present experiments was 250 ng protein per ml. At this RTA concentration five different batches of 636 mediated highly significant and selective cytotoxicity against MKN45 as previously reported (Robins *et al.*, 1990). Bispecific 636 was frequently used in control wells at up to 8 μg ml$^{-1}$ in the absence of r-RTA, and never resulted in cytotoxicity on its own.

*Influence of B chain on cytotoxicity by r-RTA and 636*

Purified RTB was completely non-toxic to cells at a concentration of 10 ng ml$^{-1}$ and was used routinely at 2.5 ng ml$^{-1}$. When combined at this dose with 250 ng ml$^{-1}$ r-RTA the resultant cytotoxicity was approximately 60% to 70% above background, indicating a synergistic interaction between the two ricin fractions. At these concentrations the presence of 636 bispecific antibody produced a modest additive increase in cytotoxicity, as shown in Figure 1. In this experiment, added RTB made the non-toxic concentration of r-RTA significantly cytotoxic for MKN45 cells. Increasing amounts of antibody also induced cytotoxicity on the part of the r-RTA, and cytotoxicity of combined r-RTA + RTB was enhanced by 636 to a degree roughly equivalent to the enhancement seen with r-RTA alone. When the antibody was used at a constant concentration of 4 μg ml$^{-1}$ and the RTB was titrated, a similar additive response was observed, up to the point where the effect of added RTB became maximal and therefore not subject to further increase (Figure 2). Thus at RTB concentrations of between 0.0001 and 0.01 ng ml$^{-1}$, which did not increase RTA cytotoxicity, a constant augmentation to about 35% cytotoxicity occurred due to 636. When RTB reached an active concentration (1 ng ml$^{-1}$) the effect of 636 was additive, until augmentation to almost 100% cytotoxicity was achieved by RTB alone at 10 ng ml$^{-1}$ as well as by RTB + 636. The antigenic specificity of this additive response is shown in Figure 3, where RTB + 636 can be seen to give greater r-RTA cytotoxicity than RTB against MKN45 cells, while there was no significant difference between RTB + 636 and RTB in the case of CEA-negative 791T cells. When r-RTA was titrated against a fixed concentration of

**Figure 1** Titration of 636 against r-RTA (250 ng ml$^{-1}$) + RTB (2.5 ng ml$^{-1}$). (□) r-RTA alone, (●) r-RTA + RTB. Cells were MKN45. Vertical bars indicate standard errors of means. RTB enhanced cytotoxicity above levels achieved with r-RTA alone, and 636 resulted in additive potentiation at all concentrations ($P < 0.001$).

**Figure 2** Titration of RTB against r-RTA (250 ng ml$^{-1}$) and 636 (4 μg ml$^{-1}$). (□) r-RTA alone, (●) r-RTA + 636. Cells were MKN45. Vertical bars indicate standard errors. 636 enhanced cytotoxicity above levels achieved with r-RTA alone, and RTB resulted in additive potentiation between 0.1 ng ml$^{-1}$ and maximum % cytotoxicity ($P<0.001$ at 1 ng ml$^{-1}$).

**Figure 3** Specificity of combined potentiation of r-RTA (250 ng ml$^{-1}$) by 636 (2.5 μg ml$^{-1}$) and titrated RTB. (■) MKN45 + 636, (●) MKN45 without antibody, (□) 791T + 636, (○) 791T without antibody. Vertical bars indicate standard errors. Cytotoxicity against both cell lines was potentiated by RTB above 0.1 ng ml$^{-1}$, but the effect of 636 alone or combined with RTB was seen only with MKN45 ($P<0.001$ comparing MKN45 + 636 with 791T + 636).

RTB (2.5 ng ml$^{-1}$) or RTB (2.5 ng ml$^{-1}$) plus 636 (8 μg ml$^{-1}$) using MKN45 cells, additive responses of 636 and RTB were again seen (Figure 4). The r-RTA was titrated from 100 ng ml$^{-1}$ downwards, and at these concentrations augmentation of cytotoxicity by 636 was less pronounced than at 250 ng ml$^{-1}$ RTA. However, RTB was more effective than 636 alone, and a mixture of 636 and RTB was the most effective treatment.

*Effect of separating sequential treatments*

In some assays RTB treatment was delayed until 4 h after the addition of r-RTA to 636-coated cells, and in these experiments a comparison was made between cultures which were washed after 30 min incubation with 636, but before addition of r-RTA, and after 4 h r-RTA incubation but before addition of RTB, and cultures which received no washing or removal of the previous reagent before application of the next in the sequence (Table I).

In these tests both 636 at 4 μg ml$^{-1}$ and RTB at 2.5 ng ml$^{-1}$ again significantly enhanced r-RTA cytotoxicity ($P<0.001$), and the combination of 636, r-RTA and RTB was more effective than treatment with 636 + r-RTA ($P<0.025$) or r-RTA + RTB ($P<0.01$). Combined 636, r-RTA and

**Figure 4** Titration of r-RTA against RTB (2.5 ng ml$^{-1}$), 636 (8 μg ml$^{-1}$), or RTB (2.5 ng ml$^{-1}$) + 636 (8 μg ml$^{-1}$), using MKN45 target cells. Mean cytotoxicity of r-RTA alone over the range 0.01–100 ng ml$^{-1}$ was −1.4%. (○) 636, (●) RTB, (□) RTB + 636. Vertical bars indicate standard errors. r-RTA cytotoxicity for MKN45 followed the pattern RTB + 636 > RTB > 636; the effects of RTB and 636 together were additive. At 1 ng ml$^{-1}$ r-RTA, RTB + 636 *vs* RTB $P<0.05$ and RTB + 636 *vs* 636 $P<0.02$; at 10 ng ml$^{-1}$ r-RTA, RTB + 636 *vs* RTB $P<0.05$ and RTB + 636 *vs* 636 $P<0.01$; at 100 ng ml$^{-1}$ r-RTA, RTB + 636 *vs* RTB $P<0.05$, RTB + 636 *vs* 636 $P<0.001$, and RTB *vs* 636 $P<0.01$.

RTB was equally effective whether RTB was added immediately after r-RTA or 4 h later, and whether or not the previous reagent was removed and the cells washed at any or all of the reagent addition steps. None of the modifications produced results significantly different from the standard procedure in which all the reagents remained present until termination of the assay. This indicates that adequate 636 binding to MKN45 could occur within 30 min, and that accumulation of sufficient r-RTA/636 complex on (or in) the target cells was completed within the next 4 h. Furthermore, the enhancement of its internalisation and subsequent cytotoxic action by RTB was not dependent on reassociation of free r-RTA and RTB in the culture medium.

*Effect of 636 on cytotoxicity of galactose-blocked ricin*

Galactose at high concentrations was inhibitory to MKN45 cells, but at 3 mg ml$^{-1}$ (0.16 mM) it was virtually non-inhibitory and was sufficiently in excess of lectin sites on the B chain to reduce the cytotoxicity of 1 ng ml$^{-1}$ whole ricin to about 5%. In the absence of galactose the 50% inhibitory concentration of ricin for MKN45 was about 0.1 ng ml$^{-1}$, and cytotoxicity at 1 ng ml$^{-1}$ was almost 100%. Titration of 636 against galactose-blocked ricin at 1 ng ml$^{-1}$ produced increasing cytotoxicity against MKN45, until at 4 μg ml$^{-1}$ antibody it was restored to about 80% of the level seen with native ricin (Figure 5). Purified RTB (2.5 ng ml$^{-1}$) was similarly blocked with 3 mg ml$^{-1}$ galactose and tested in combination with r-RTA (250 ng ml$^{-1}$) and titrated 636, as also shown in Figure 5. The previously demonstrated ability of RTB to augment r-RTA cytotoxicity was abolished by galactose, but full cytotoxic activity was restored in the presence of increasing amounts of 636.

The relative cytotoxic activities of r-RTA, blocked ricin and r-RTA + blocked RTB in the absence of 636 and in the presence of the highest 636 concentration tested (8 μg ml$^{-1}$) are compared in Figure 6. This comparison puts into perspective the role of B chain in facilitating A chain cytotoxicity. Targeting with 636 antibody was effective with r-RTA alone, but the presence of an adequate amount of blocked B chain (either as part of intact ricin or as added RTB at 1% of the r-RTA concentration) significantly enhanced the efficiency of r-RTA targeting.

### Discussion

Bispecific antibodies capable of targeting toxic moieties to tumour cells *in vitro* have been produced by chemical hydro-

**Figure 5** Effect of 636 on cytotoxicity of galactose-blocked ricin (1 ng ml$^{-1}$) and r-RTA (250 ng ml$^{-1}$) + galactose-blocked RTB (2.5 ng ml$^{-1}$) against MKN45 cells. (□) blocked ricin, (●) r-RTA + blocked RTB. Vertical bars indicate standard errors. Both responded highly significantly ($P<0.001$ at or above 0.25 μg ml$^{-1}$ 636).

**Figure 6** Comparison of augmented cytotoxicity against MKN45 using 8 μg ml$^{-1}$ 636 antibody. (■) r-RTA (250 ng ml$^{-1}$), (□) galactose-blocked ricin (1 ng ml$^{-1}$), (▨) r-RTA (250 ng ml$^{-1}$) + galactose-blocked RTB (2.5 ng ml$^{-1}$). Vertical bars on the histograms indicate standard errors. Cytotoxicity of all reagents was augmented by 636 compared with background ($P<0.001$), but augmentation was greater with blocked ricin or r-RTA + blocked RTB than with RTA alone ($P<0.001$).

lysis and reconstitution of two parental antibodies (Raso & Griffin, 1981; Glennie *et al.*, 1988) and by production of hybridomas secreting monoclonal antibodies with dual specificity (Webb *et al.*, 1985, 1986; Corvalan & Smith, 1987; Pimm *et al.*, 1990). In studies in which the toxic molecule was a plant toxin such as gelonin (Glennie *et al.*, 1988) or RTA (Webb *et al.*, 1985, 1986), the reported efficiency of targeting by bispecific antibodies was comparable with that commonly observed with conventional immunotoxins in which toxin and anti-tumour monoclonal antibody are linked chemically (Davies & Crumpton, 1982; Moller, 1982). In the case of bispecific antibody 636, significant cytotoxicity against MKN45 could be achieved at an RTA concentration as low as $4 \times 10^{-9}$ M (Robins *et al.*, 1990), which compares with independent experiments performed with chemically prepared conjugates between RTA and a different CEA-specific antibody (228), in which the most active conjugates killed 50% of MKN45 cells at a concentration of about $1.5 \times 10^{-9}$ M RTA (Byers *et al.*, 1988). The augmentation of RTA activity by 636 was specific for CEA expression cells, and was dependent upon the dual specificity of the antibody; parental anti-CEA and anti-RTA monoclonal antibodies had no effect at all (Robins *et al.*, 1990).

The cytotoxicity of RTA immunotoxins can be enhanced in several ways, notably the addition of lysosomotropic agents such as amines or proton ionophores or administration of RTB. Lysosomotropic agents appear to act by raising the pH of endosomes (Sandvig & Olsnes, 1982; Wileman *et al.*, 1985). This can result in the increased release of ricin following internalisation of ricin-ligand complexes, and it is possible that RTA is released from internalised immunotoxins in a similar manner. RTB can be administered in free form, or conjugated to the same targeting antibody as the RTA immunotoxin or any antibody recognising the immunotoxin conjugate (McIntosh *et al.*, 1983; Vitetta *et al.*, 1983, 1984), and is thought to aid cellular incorporation of immunotoxin by facilitating translocation through the cell membrane (McIntosh & Thorpe, 1984). In cases where the targeted antigen is rapidly and efficiently internalised by the cell the lack of B chain may be advantageous but non-essential, but there are instances in which RTA immunotoxin is not readily incorported and the transport function of the B chain then becomes of major importance (McIntosh *et al.*, 1983; Eccles *et al.*, 1987). Hence the continued interest in facilitation of immunotoxin uptake by extraneous B chain.

The reason for removing the B chain before conventional immunotoxin preparation is that its lectin site (binding to cellular galactose residues) is quantitatively much more effective at binding to cells and delivery of A chain than a conjugated antibody, and this would overcome and negate any selectivity contributed by the antibody. It is possible, however, to block lectin activity of whole ricin conjugates with excess lactose or galactose, or to select intrinsically blocked whole ricin conjugate molecules by chromatography (Thorpe & Ross, 1982; Gregg *et al.*, 1987; Cattel *et al.*, 1988). Galactose blocking can be very effective in aiding selectivity of ricin-antibody conjugates *in vitro*, and a galactose-blocked ricin anti-iodiotype antibody conjugate has been tested *in vivo* against a guinea pig B cell leukaemia line (Gregg *et al.*, 1987). The blocked ricin conjugate was therapeutic at 100-fold lower concentration than an equivalent RTA immunotoxin, but was also 100 times more toxic at equimolar doses, so that the overall therapeutic index was the same for both reagents.

The mechanism of action of RTB in potentiating RTA uptake has not been established. One assumption is that A and B chains reassociate to form intact toxin (McIntosh & Thorpe, 1984) which then presumably acts at the target site. In the present studies r-RTA was used at a standard concentration of about $8 \times 10^{-10}$ M, and RTB at a concentration of only 1% of this, so most of the r-RTA could not be involved in reassociation. If, however, all the RTB had reassociated with r-RTA on an equimolar basis, the resultant 'reconstituted ricin' could have been present in a quantity more than adequate to kill 100% of the target cells. Further titration of RTB (Figure 2) or r-RTA (Figure 4) resulted in loss of cytotoxicity and this may have been due to the diminished potential for adequate reassociation. However, if full reconstitution had taken place under the standard conditions one would expect the resultant cytotoxicity to over-ride any augmenting effect of the 636 antibody. The fact that the cell kill was low enough to detect 636 augmentation of r-RTA + RTB cytotoxicity at the concentrations used in Figures 1 and 2, and that it was specific for CEA-positive cells (Figure 3) suggests that complete reassociation did not occur. Nevertheless, it is probable that partial reassociation is involved in potentiation of cytotoxicity, and the maximal cytotoxicity values observed in Figures 2 and 3 at the highest RTB concentration might be accounted for by the accumulation of sufficient recombined toxin. The experiments in which excess 636 and r-RTA were removed before RTB was applied to the cells (Table I) suggests that if reassociation is necessary, it can occur at the target cell site, and the formation of free ricin in the supernatant culture medium is not required. RTB augmentation of cytotoxicity was equally high in washed cells as in those which were left in continuous contact with 636, r-RTA and RTB for a further 60 h.

Opinion is divided on whether or not an active lectin binding site is required for augmentation to occur. This cannot satisfactorily be resolved by studying chemically linked immunotoxins, because the need to avoid lectin activity is fundamental to their design, and in published studies of the effect of RTB or RTB conjugates on RTA immunotoxins the B chain lectin site has not been blocked (McIntosh *et al.*, 1983; Eccles *et al.*, 1987; Vitetta *et al.*, 1983, 1984). The experiments shown in Figure 5, however, address this question. Whole ricin inactivated by blocking its B chain lectin site with galactose was rendered highly cytotoxic by the addition of 636 bispecific antibody, and there is no doubt that the B chain played an important role in this because the ricin was used at approximately 20 times lower molarity than the lowest concentration of RTA required for 636-mediated cytotoxicity (Robins *et al.*, 1990). A mixture of intact RTB and r-RTA at the concentrations shown in highly cytotoxic, presumably because the RTB reassociates with sufficient r-RTA and mediates its entry into the cell by binding via the lectin site. When the lectin site was blocked with galactose as in Figure 5, cytotoxicity was lost owing to the lack of binding ability. However, addition of 636 to induce binding of r-RTA to the cells restored the augmented level of cytotoxicity characteristic or r-RTA + unblocked RTB + 636. These results suggest that the B chain translocation function is still active when the lectin site is blocked, and therefore that the lectin site is not involved. This is in agreement with results of studies using intrinsically blocked whole ricin-antibody conjugates (Cattel *et al.*, 1988). A blocked ricin immunotoxin had cytotoxic activity as high as that of an unblocked ricin conjugate, but was much more selective than the unblocked reagent. An RTA immunotoxin showed the same high selectivity, but was much less toxic than the blocked ricin conjugate.

The studies reported here demonstrate that a bispecific monoclonal antibody can provide an effective means of targeting r-RTA *in vitro*, and that the cytotoxicity observed is able to be potentiated by the addition of RTB. Moreover, the RTB is not required to possess an active lectin site. On theoretical grounds, there may be advantages to such a combination in comparison with a conventional immunotoxin when applied *in vivo* in a therapeutic context. The bispecific antibody may localise at the tumour site more efficiently than an RTA-antibody or blocked ricin-antibody conjugate, which is likely to be rapidly cleared from the circulation (Simmons *et al.*, 1986; Byers *et al.*, 1987). Subsequently administered RTA may then be trapped by the pre-localised antibody in higher quantities, before elimination, than could be delivered as an immunotoxin, particularly if recombinant RTA (lacking carbohydrate side chains) is used. Also, the possibility of using lectin-inactivated B chain to enhance the effect of localised A chain may be a safer approach than using intact B chain which could conceivably recombine with A chain to form active ricin. These potential advantages now require to be evaluated in a therapeutic model.

The provision of reagents by the XOMA Corporation and Professor M. Lord is gratefully acknowledged. This work was supported by the Cancer Research Campaign, London, UK.

### References

BYERS, V.S., PAWLUCZYK, I., BERRY, N. & 5 others (1988). Potentiation of anti-carcinoembryonic antigen immunotoxin cycotoxicity by monoclonal antibodies reacting with co-expressed carcinoembryonic antigen epitopes. *J. Immunol.*, **140**, 4050.

BYERS, V.S., PIMM, M.V., PAWLUCZYK, I.Z.A., LEE, H.M., SCANNON, P.J. & BALDWIN, R.W. (1987). Biodistribution of ricin toxin A chain-monoclonal antibody 791T/36 immunotoxin and influence of hepatic blocking agents. *Cancer Res.*, **47**, 5277.

CATTEL, L., DEPRINO, L., BRUSA, P., DOSIO, F., COMOGLIO, P. & PRAT, M. (1988). Comparison of blocked and non-blocked ricin-antibody immunotoxins against gastric carcinoma and colorectal adenocarcinomas cell lines. *Cancer Immunol. Immunother.*, **27**, 233.

CORVALAN, J.R.F. & SMITH, W. (1987). Construction and characterisation of a hybrid-hybrid monoclonal antibody recognising both carcinoembryonic antigen and vinca alkaloids. *Cancer Immunol. Immunother.*, **24**, 127.

DAVIES, A.J.S. & CRUMPTON, M.J. (1982). (eds). Experimental approaches to drug targeting. *Cancer Surveys*, **1**, 347.

ECCLES, S.A., MCINTOSH, D.P., PURVIES, H.P. & 5 others (1987). An ineffective monoclonal antibody-ricin A chain conjugate is converted to a tumouricidal agent *in vivo* by subsequent administration of ricin B chain. *Cancer Immunol. Immunother.*, **24**, 37.

EIKLID, K., OLSNES, S. & PIHL, A. (1980). Entry of lethal doses of abrin, ricin and modeccin into the cytosol of HeLa cells. *Exp. Cell Res.*, **126**, 321.

FITZGERALD, D. & PASTAN, I. (1989). Targeted toxin therapy for the treatment of cancer. *J. Natl Cancer Inst.*, **81**, 1455.

GLENNIE, M.J., BRENNAND, D.M., BRYDEN, F. & 4 others (1988). Bispecific F(ab')$_2$ antibody for the delivery of saporin in the treatment of lymphoma. *J. Immunol.*, **141**, 3662.

GREGG, E.O., BRIDGES, S.H., YOULE, R.J. & 5 others (1987). Whole ricin and recombinant A chain idiotype-specific immunotoxins for immunotherapy of the guinea pig $L_2C$ B cell leukaemia. *J. Immunol.*, **138**, 4502.

MCINTOSH, D.P., EDWARDS, D.C., CUMBER, A.J. & 4 others (1983). Ricin B converts a non-toxic antibody-ricin A chain conjugate into a potent and specific cytotoxic agent. *FEBS Lett.*, **164**, 17.

MCINTOSH, D.P. & THORPE, P.E. (1984). Role of the B chain in the cytotoxic action of antibody-ricin and antibody-abrin conjugates. In Gregoriadis, G., Poste, G., Senior, J. & Trouet, A. (eds) *Receptor-Medicated Targeting of Drugs*. Plenum Press: New York, London, p. 105.

MOLLER, G. (1982). (ed.) Antibody carriers of drugs and toxins in tumor therapy. *Immunol. Revs.*, **62**.

PIMM, M.V., ROBINS, R.A., EMBLETON, M.J. & 4 others (1990). A bispecific monoclonal antibody against methotrexate and a human tumour associated antigen augments cytotoxicity of methotrexate-carrier conjugate. *Br. J. Cancer*, **61**, 508.

PRICE, M.R., EDWARDS, S.S., JACOBS, E., PAWLUCZYK, I., BYERS, V.S. & BALDWIN, R.W. (1987). Mapping of monoclonal antibody-defined epitopes with carcinoembryonic antigen, CEA. *Cancer Immunol. Immunother.*, **25**, 10.

RASO, V. & GRIFFIN, T. (1981). Hybrid antibodies with dual specificity for delivery of ricin to immunoglobulin bearing target cells. *Cancer Res.*, **41**, 2073.

ROBINS, R.A., EMBLETON, M.J., PIMM, M.V. & 4 others (1990). Bispecific antibody which binds to carcinoembryonic antigen and ricin toxin A chain is cytotoxic for gastrointestinal tract tumour cells. *J. Natl Cancer Inst.* (in press).

SANDVIG, K. & OLSNES, S. (1982). Entry of the toxic proteins abrin, modeccin, ricin and diptheria toxin into cells. II. Effect of pH, metabolic inhibitors and ionophores, and evidence for toxin penetration from endocytic vesicles. *J. Biol. Chem.*, **257**, 7504.

SIMMONS, B.M., STAHL, P.D. & RUSSELL, J.H. (1986). Mannose receptor-mediated uptake of ricin toxin and ricin A chain by macrophages. *J. Biol. Chem.*, **261**, 7912.

THORPE, P.E., DETRE, S.I., FOXWELL, B.M.J. & 5 others (1985). Modification of the carbohydrate in ricin with metaperiodate-cyanoborohydride mixtures: effects on toxicity and *in vivo* distribution. *Eur. J. Biochem.*, **147**, 197.

THORPE, P.E. & ROSS, W.C.J. (1982). The preparation and cytotoxic properties of antibody-toxin conjugates. *Immunol. Revs.*, **62**, 119.

VITETTA, E.S., CUSHLEY, W. & UHR, J.W. (1983). Synergy of ricin A-chain containing immunotoxins and ricin B-chain immunotoxins in *in vitro* killing of neoplastic human B cells. *Proc. Natl Acad. Sci. USA*, **80**, 6332.

VITETTA, E.S., FULTON, R.J. & UHR, J.W. (1984). Cytotoxicity of a cell-reactive immunotoxin containing ricin A-chain is potentiated by an immunotoxin containing ricin B chain. *J. Exp. Med.*, **160**, 341.

WEBB, K.S., LIBERMAN, S.N., WARE, J.L. & WALTHER, P.J. (1986). *In vitro* synergism between hybrid immunotoxins and chemotherapeutic drugs: relevance to immunotherapy of prostate carcinoma. *Cancer Immunol. Immunother.*, **21**, 100.

WEBB, K.S., WARE, J.L., PARKS, S.F., WALTHER, P.J. & PAULSON, D.F. (1985). Evidence for a novel hybrid immunotoxin recognising ricin A chain by one antigen combining site and a prostate-restricted antigen by the remaining antigen combining site: potential for immunotherapy. *Cancer Treatment Rep.*, **69**, 663.

WILEMAN, T., HARDING, C. & STAHL, P. (1985). Receptor-mediated endocytosis. *Biochem. J.*, **232**, 1.